# Multiple Solutions to the Same Problem: Utilization of Plausibility and Syntax in Sentence Comprehension by Older Adults with Impaired Hearing

**DOI:** 10.3389/fpsyg.2016.00789

**Published:** 2016-05-30

**Authors:** Nicole M. Amichetti, Alison G. White, Arthur Wingfield

**Affiliations:** Volen National Center for Complex Systems, Brandeis University, WalthamMA, USA

**Keywords:** sentence comprehension, plausibility, adult aging, hearing impairment, working memory

## Abstract

A fundamental question in psycholinguistic theory is whether equivalent success in sentence comprehension may come about by different underlying operations. Of special interest is whether adult aging, especially when accompanied by reduced hearing acuity, may shift the balance of reliance on formal syntax vs. plausibility in determining sentence meaning. In two experiments participants were asked to identify the thematic roles in grammatical sentences that contained either plausible or implausible semantic relations. Comprehension of sentence meanings was indexed by the ability to correctly name the agent or the recipient of an action represented in the sentence. In Experiment 1 young and older adults’ comprehension was tested for plausible and implausible sentences with the meaning expressed with either an active-declarative or a passive syntactic form. In Experiment 2 comprehension performance was examined for young adults with age-normal hearing, older adults with good hearing acuity, and age-matched older adults with mild-to-moderate hearing loss for plausible or implausible sentences with meaning expressed with either a subject-relative (SR) or an object-relative (OR) syntactic structure. Experiment 1 showed that the likelihood of interpreting a sentence according to its literal meaning was reduced when that meaning expressed an implausible relationship. Experiment 2 showed that this likelihood was further decreased for OR as compared to SR sentences, and especially so for older adults whose hearing impairment added to the perceptual challenge. Experiment 2 also showed that working memory capacity as measured with a letter-number sequencing task contributed to the likelihood that listeners would base their comprehension responses on the literal syntax even when this processing scheme yielded an implausible meaning. Taken together, the results of both experiments support the postulate that listeners may use more than a single uniform processing strategy for successful sentence comprehension, with the existence of these alternative solutions only revealed when literal syntax and plausibility do not coincide.

## Introduction

A critical feature of spoken language is its rapidity, with everyday speech rates often exceeding 180 to 200 words per minute (Stine et al., 1990). The fact that spoken sentences can be successful comprehended in spite of this rapid input rate raises the question of whether listeners may necessarily engage in a fully exhaustive word-by-word analysis of a sentence to determine its meaning. That is, rather than building a detailed and compete representation of the utterance, listeners may under some circumstances analyze the lexical input to a level of detail that is just “good enough” to extract the sentence meaning, with this especially so when the listener is faced with sentences that express their meaning with relatively complex syntactic structures (Ferreira et al., 2002; Ferreira, 2003; Christianson et al., 2006; Ferreira and Patson, 2007). Listeners must also comprehend sentences that contain ungrammatical or underspecified structures, a common occurrence in everyday communication (Goldman-Eisler, 1968; Elsness, 1984; Thompson and Mulac, 1991). In such cases it has been argued that comprehension is accomplished based on probabilistic inferences and plausibility substituting for operations represented in formal hierarchical syntactic processing models (e.g., Ferreira, 2003; Padó et al., 2009; Frank and Bod, 2011; Gibson et al., 2013).

The process we are describing can be referred to as *shallow processing*, a processing strategy in which the meaning of a sentence is rapidly inferred based on word order and thematic plausibility (Ferreira, 2003). Because we live in a relatively predictable and usually plausible world, this processing heuristic will ordinarily be successful. It will fail only in those circumstances when a sentence conveys an unexpected or unlikely meaning. It has been suggested by Rönnberg et al. (2013) that when a listener is under time pressure, and willing to accept the gist of a message, a thorough analysis might not take place. Indeed, it has been argued that detailed and time-consuming lexical and syntactic analyses of an utterance may be an exception, rather than the rule (Ferreira, 2003; Ferreira and Patson, 2007).

This position argues against traditional assumptions of a single “optimal” model of sentence processing that underlies successful comprehension. Rather, it is possible that a range of processing heuristics, ranging from relatively more shallow to more exhaustive word-by-word processing, will produce similar consequences under usual, but not all, circumstances. Broadly defined, this is a position in tune with a developing recognition in modern neurobiology that is showing that a range of circuit parameters are “good enough” to yield the same output, although not all solutions may be equally robust to potential perturbations (Marder, 2011; Tang et al., 2012).

In an analogous manner, we argue that uniform success in sentence comprehension need not imply that each incidence of successful comprehension has been achieved by the same cognitive route. This can be revealed when listeners are presented with sentences that contain an implausible meaning. This circumstance appeared, for example, when Ferreira (2003) presented university students with sentences that expressed a plausible or an implausible meaning with an active-declarative syntactic form (e.g., “The dog bit the man”; “The man bit the dog”) or plausible or implausible sentences with a less common passive construction (e.g., “The man was bit by the dog”; “The dog was bit by the man”). When asked to identify the thematic roles in such sentences (who did the biting; who was bit), listeners were more likely to focus on plausibility (responding that the dog bit the man) when the meaning was conveyed with the less canonical passive syntactic structure. In such cases listeners’ use of a shallow processing heuristic rather than a fully exhaustive word-by-word analysis will be revealed when thematic plausibility over-rides meaning based on the literal syntax of a sentence. This issue may take on special importance in the context of adult aging, where both working memory resources and hearing acuity typically show some degree of decline.

## The Special Challenges of Adult Aging and Hearing Impairment

Although hearing loss is a common accompaniment of adult aging, it has primarily been considered as an independent issue in cognitive aging research. We now know that there are subtle but important effects of reduced hearing acuity beyond simply missing or misidentifying individual words in a spoken message. That is, when speech is degraded, either due to reduced hearing acuity or due to acoustic masking, the cognitive effort needed for successful perception can take a toll on both comprehension and memory for spoken materials (cf., Rabbitt, 1968, 1991; Surprenant, 1999, 2007; Pichora-Fuller, 2003; McCoy et al., 2005; Wingfield et al., 2006; Piquado et al., 2010, 2012). Importantly, these effects appear even when it can be demonstrated that the speech itself has passed a threshold of audibility.

It is known that older adults have more difficulty than their younger adult counterparts in understanding sentences with complex syntactic structures (Wingfield et al., 2003, 2006). This has been attributed to increased working memory demands required for comprehension of such sentences that place older adults at a special disadvantage (Carpenter et al., 1994). Combined with an age-related hearing impairment adding to the processing challenge, a shift toward a processing heuristic that is adequate for comprehension, rather than one that engages a more resource-demanding, fully exhaustive syntactic analysis, might be expected to lead older adults to the more frequent use of plausibility rather than to the literal syntactically determined meaning of an utterance. Thus, to the extent that successful speech recognition in the face of hearing loss may draw resources needed for processing the sentence meaning, shallow processing may be more likely for older adults with hearing impairment than for young adults or for older adults with good hearing acuity.

We report the results of two experiments designed to test this hypothesis. The first experiment was patterned after Ferreira (2003), although with older as well as younger adults. Following Ferreira (2003), sentences were heard with either plausible or implausible meanings expressed with either an active-declarative structure or a less canonical passive structure. Our question was whether plausibility would be more likely to over-ride the literal syntactically determined meaning for older adults as compared to young adults. This first experiment was intended to define the lower boundaries of a potential interaction between adult aging and a plausibility bias, as the syntactic contrast between active-declarative and passive structures is a relatively mild one (see data in Gibson et al., 2013) and the older adults for this experiment would be especially selected for good hearing acuity.

In Experiment 2 comprehension was assessed when the processing challenge was further increased in two ways. First, the syntactic contrast would be between sentences with a subject-relative (SR) embedded clause structure and a much more complex object-relative (OR) embedded clause structure. This syntactic contrast was chosen because the comprehension of OR sentences is known to produce significantly greater processing demands than SR sentences (Ferreira et al., 1996; Just et al., 1996; Gibson, 1998; Cooke et al., 2002; Wingfield et al., 2006; Peelle et al., 2010; Staub, 2010). Second, the experiment was conducted with two groups of older adults: one group who had good hearing acuity for their ages and another group with a bilateral mild-to-moderate hearing loss, the most common degree of loss among older adults with hearing impairment (Morrell et al., 1996). Our question was whether the combined challenge of complex syntax combined with perceptual effort due to a hearing impairment, would increase the likelihood of a listener conducting a more shallow analysis of the speech input. Such a processing strategy would be revealed by a comprehension response to an implausible sentence (i.e., one with an unlikely meaning), that relies on plausibility rather than on its literal syntactically based meaning.

## Experiment 1

### Method

#### Participants

Participants were 24 young adults (2 men, 22 women) ranging in age from 18 to 30 years (*M* = 20.2 years, *SD* = 2.4) and 24 older adults (7 men, 17 women) ranging in age from 66 to 82 years (*M* = 75.1 years, *SD* = 4.2). The young adults were university students and staff and the older participants were healthy community-dwelling volunteers. To insure that any age decrements would not be attributable to an accidental difference in vocabulary knowledge all participants were screened with the Shipley Vocabulary Test (Zachary, 1991). As is common for healthy older adults (Kempler and Zelinski, 1994; Verhaeghen, 2003), the older adults in this study had an advantage in terms of vocabulary knowledge [*M* younger = 13.3, *SD* = 2.0; *M* older = 17.0, *SD* = 2.3; *t*(46) = 5.99, *p* < 0.001]. All participants reported themselves to be in good health, with no self-reported history of stroke, Parkinson’s disease, or other neurologic involvement that might compromise their ability to perform the research task. All participants reported themselves to be native speakers of American English.

Audiometric evaluation was carried out for each participant using a GSI 61 clinical audiometer (Grason-Stadler, Inc., Madison, WI, USA) by way of standard audiometric techniques in a sound-attenuated testing room (Harrell, 2002). The young adults had a mean better-ear pure tone threshold average (PTA) of 8.0 dB HL (*SD* = 4.5) averaged over 500, 1,000, 2,000, and 4,000 Hz. The older adults had a mean better-ear PTA (500, 1,000, 2,000, and 4000 Hz) of 23.2 dB HL (*SD* = 6.5). Participants who demonstrated unbalanced hearing (more than a 15 dB difference between ears in one or more frequencies) were excluded from participation.

Although elevated relative the young adults, *t*(46) = 9.42, *p* < 0.001, the older adults’ thresholds fell within or close to a range typically considered to be clinically normal for speech (PTA < 25 dB HL; Katz, 2002). None of the older participants wore hearing aids on a regular basis, and all testing was conducted unaided. Written informed consent was obtained from all participants according to a protocol approved by the Brandeis University Institutional Review Board.

#### Stimuli

A total of 16 active-declarative sentences, patterned after Ferreira (2003; Experiment 1), were constructed to contain an agent of an action and a recipient of that action. Active-declarative sentences represent a typical noun-verb-noun (NVN) structure, in which the first noun is the agent of the action. From each of these active-declarative sentences we constructed an additional 16 sentences with the same meaning but with this meaning expressed with a less canonical passive structure, in which the second noun is the agent of the action.

Four versions of each sentence were constructed: an active-declarative version with a plausible action (e.g., “The eagle attacked the rabbit”), an active-declarative version with the agent and recipient switched to yield a less likely (implausible) action (e.g., “The rabbit attacked the eagle”), a passive sentence structure with a plausible action (e.g., “The rabbit was attacked by the eagle”), and a passive version with an implausible action (e.g., “The eagle was attacked by the rabbit”). This resulted in 64 experimental sentences: 16 base sentences consisting of a unique set of nouns and action verbs with four versions of each.

In addition to these 64 experimental sentences (16 base sentences × 4 versions of each), 72 filler sentences were constructed to avoid a uniform pattern of plausible and implausible non-reversible sentences. Two-thirds of the fillers contained an active or passive construction but in which the agent and recipient could be exchanged without affecting plausibility (e.g., “The boy thanked the girl”; “The girl thanked the boy”). Other fillers were constructed that were non-reversible (e.g., “The man walked across the street”; “The bird was bright red”). Each participant heard 36 fillers (24 reversible fillers, and 12 non-reversible fillers). These fillers did not form part of the experimental analyses.

The experimental and filler sentences were recorded onto computer sound files by a female speaker of American English at a natural speaking rate of approximately 165 words per minute (wpm) with normal prosody using Sound Studio v2.2.4 software (Macromedia, Inc., San Francisco, CA, USA) that digitized (16-bit) at a sampling rate of 44.1 kHz. Recordings were equalized within and across sentence types for root-mean-square (RMS) intensity using MATLAB (MathWorks, Natick, MA, USA).

#### Procedure

Each participant heard a total of 64 experimental sentences, 16 active-plausible, 16 active-implausible, 16 passive-plausible, and 16 passive-implausible. No version of any base sentence (a particular combination of nouns and action verb) was heard more than once by any participant, with the particular base sentence heard in each of its versions counterbalanced across participants such that, by the end of the experiment, each base sentence had been heard in each of its versions an equal number of times. Stimuli were presented in a mixed-list design, with experimental sentences and filler sentences intermingled in a pseudo-random order across lists. This resulted in a total of 100 sentences heard by each participant.

Participants were told that following each sentence there would be a 250 ms pause, followed by a spoken probe question. For the experimental sentences and the reversible filler sentences participants were asked to name aloud either the agent or the recipient of the action, in the form of, “Who was the do-er?” or “Who was the receiver?” Participants were asked to give their responses aloud as accurately as possible. Sentences and probe questions were also counterbalanced, such that, by the end of the experiment, each of the experimental sentences and reversible fillers were followed an equal number of times by agent and recipient probes. Probe questions for the non-reversible filler sentences were “What was the color?” or “What was the action?” (Ferreira, 2003).

Participants were tested individually in a sound-attenuated testing room, with stimuli presented binaurally through calibrated Eartone 3A insert earphones (E-A-R Auditory Systems, Aero Company, Indianapolis, IN, USA), via a GSI-61 audiometer (Grason-Stadler, Madison, WI, USA) at 65 dB HL. Participants’ responses were recorded for later accuracy scoring. The main experiment was preceded by a brief practice session to familiarize participants with the task and the sound of the speaker’s voice. This session consisted of eight active and passive form sentences of similar length as the test sentences. None these sentences were used in the main experiment.

##### Audibility check

A pretest was conducted in order to insure that the speech materials would be audible to both the young and older adult participants. One- and two-syllable common nouns were presented one at a time at the same intensity level as would be used for the main experiment. After the presentation of each word participants were asked to repeat the word just heard. All participants’ report accuracy was above a pre-determined cutoff criterion of 90% accuracy, with the young adults having a mean accuracy of 98.7% words correct and the older adults 97.9% words correct.

### Results

**FIGURE 1:**
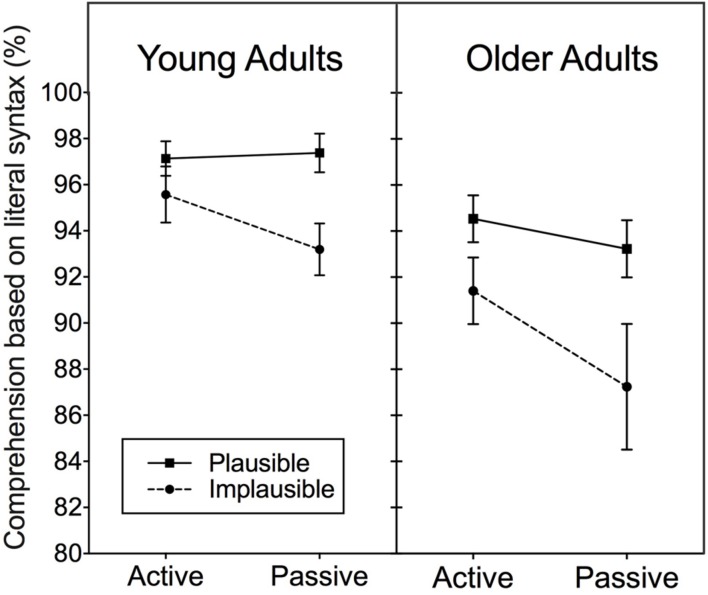
**Percentage of comprehension responses based on literal syntax represented in active and passive sentences when the sentence meanings were plausible or implausible.** Data for young and older adults are shown in the left and right panels, respectively. Error bars represent one standard error.

The left panel of **Figure 1** shows the mean percentage of times that the young adults used the literal syntax to determine who was the agent or the recipient of the action for plausible and implausible experimental sentences in which the meaning was expressed with an active or passive syntactic structure. The right panel shows these data for the older adults. There was no significant difference in response accuracy depending on whether the agent or recipient of the action was requested. For all analyses data were thus collapsed across both types of question probes.

The data shown in **Figure 1** were analyzed with a 2 (Plausibility: plausible, implausible) × 2 (Age: young, older) × 2 (Syntactic complexity: active, passive) mixed-design analysis of variance (ANOVA), with syntax and plausibility as within-participants variables and age as a between-participants variable. As can be seen in **Figure 1**, both participant groups’ responses were more likely to be consistent with the literal syntax in plausible than in implausible sentences, as confirmed by a significant main effect of plausibility, *F*(1,46) = 17.54, *p* < 0.001, $\eta_{p}^{2}$ = 0.28. There was also a significant main effect of age, *F*(1,46) = 11.95, *p* < 0.01, $\eta_{p}^{2}$ = 0.21. There was a marginal effect of syntactic complexity, *F*(1,46) = 3.46, *p* = 0.069, $\eta_{p}^{2}$ = 0.07. None of the interactions reached significance, consistent with the general similarity in patterns for both age groups.

To look more closely at the nature of these patterns subsidiary 2 (Plausibility) × 2 (Syntactic complexity) repeated measures ANOVAs were conducted separately for each participant group. Although the main effect of plausibility was significant for both groups (young adults, *p* < 0.05; older adults, *p* < 0.01), the appearance in **Figure 1** of differentially fewer responses following the literal syntax in implausible passive sentences than in implausible active sentences was not supported either a significant main effect of syntax nor a significant Syntax × Plausibility interaction for either participant group (*p*’s > 0.05). Planned comparisons did give some support for such an interaction. For the young adults a significant difference appeared between plausible and implausible passive sentences, *t*(1,23) = 3.11, *p* < 0.05, but not for the active sentences, [*t*(1,23) = 1.14, *p* = 0.27]. A similar pattern was shown for the older adults, with a significant difference appearing between plausible and implausible sentences for the passive sentences, *t*(1,23) = 2.57, *p* < 0.05 and a marginal difference between plausible and implausible sentences for the active sentences *t*(1,23) = 1.86, *p* = 0.07. It can be noted that for plausible active sentences both age groups’ accuracy was above 95% correct comprehension with the younger and older adults differing by only 2.6% points, *t*(46) = 2.06, *p* = 0.054.

Although there is a suggestion of a differential increase in reliance on plausibility for sentences with a passive structure compared to those with an active structure, it can be seen that the effect is a weak one. This is consistent with other studies (e.g., Gibson et al., 2013), that have shown a small or absent effect on comprehension responses for implausible active vs. passive sentences. In the present experiment, for example, the difference between responses based on literal syntax in implausible active vs. passive sentences amounting to only a 2.4% point difference for the young adults and a 4.7% point difference for the older adults, with neither difference approaching significance.

### Discussion

The results of Experiment 1 show that when the literal syntax of a sentence would imply an implausible meaning, older adults were less likely than their young adult counterparts to follow the meaning expressed by the literal syntax (See Obler et al., 1991, for supportive data). Although our focus is on comprehension, our findings are consistent with results from studies of verbal memory that have shown that older adults perform as well as young adults when memory probes for studied passages are plausible, but more poorly than young adults when they are implausible (e.g., Reder et al., 1986). Analogous to arguments for plausibility effects in sentence comprehension, Reder et al. (1986) suggested that older adults may employ a plausibility-based strategy because it is less resource-demanding than decisions based on specific passage details. Here we suggest that older adults tend to give heavier weight to plausibility than to the literal content in sentence comprehension when the two are in conflict as a way of conserving reduced working memory resources (See Connell and Keane, 2006, for a discussion of plausibility as a cognitive shortcut in memory retrieval).

Early syntax-based models of sentence processing (Miller and Chomsky, 1963), that largely replaced even earlier expectancy-based Markov models of language (Miller, 1952), predicted that comprehension of sentences in a passive form would be more demanding than active sentences because, for understanding, listeners would have to decompose passive sentences into their active form from which they were assumed to be derived (e.g., Miller, 1962). [In a Markov model a particular sequence of symbols (e.g., words, musical notes) is determined solely by their statistical probability based on prior events].

Although theoretical accounts of the relation between active and passive sentences have subsequently evolved (see Ratner et al., 1993, pp. 16–27, for a review of this evolution), early studies showed poorer comprehension and recall of passive sentences than active sentences (e.g., Miller, 1962; Savin and Perchonock, 1965). These studies, offered in support of a derivational theory of sentence complexity, however, were not without criticism on methodological grounds (cf., Wearing, 1970; Boakes and Lodwick, 1971).

It is the case that passive sentence structures are less likely to be encountered in everyday listening experience than active-declarative sentences. For example, an analysis of the types of sentences heard in a British sample of everyday discourse found that simple declarative sentences were by far the most commonly used grammatical forms, accounting for 70–80% of the spoken sentences in the sample. By contrast, passives were encountered in only 0.7–11% of everyday spoken discourse (Goldman-Eisler and Cohen, 1970). It may thus be the case that a listener’s expectation of hearing an active-declarative sentence, in which the first noun is the agent of the action, must be rejected for successful comprehension. Such an argument has been made by Yoon et al. (2015). (See Novick et al., 2005, for an analogous account of the comprehension difficulty for passive sentences encountered in patients with Broca’s aphasia). We found young adults responded to plausibility more frequently than literal syntax for passive sentences, similarly to Ferreira (2003) who also tested young adults. In experiment 1 we showed this same effect also held for older adults although not to a differentially greater degree than the young. It should be noted, however, that the size of the effect was small for both age groups suggesting that both the young and older adults in our study were adept at dealing with this frequency-based violation.

Although the effect of our syntactic manipulation was small, plausibility of the utterance had an impact on performance. In the case of plausible sentences one cannot tell whether the listener is basing his or her comprehension on the literal syntax of the sentence or the plausibility, as the two coincide. The test comes with sentences where the literal syntax and semantic plausibility are in conflict. When this occurred, the incidence of sentence comprehensions that followed the literal syntax was reduced. Even for the older adults, however, comprehension responses based on the literal syntax predominated for both syntactic forms examined.

## Experiment 2

In Experiment 1 all of the older adults had good hearing acuity for their ages. This raises the question of whether the extra processing load induced by reduced hearing acuity, as is more typical of older adults (Morrell et al., 1996), might increase reliance on a resource-conserving strategy represented by shallow processing, and especially so when the sentence meaning is expressed with a more challenging syntactic manipulation than used in Experiment 1.

In Experiment 2 we thus examined effects of hearing acuity on comprehension responses to determine whether perceptual effort consequent to reduced hearing acuity will amplify the shift to a plausibility-weighted algorithm, or alternatively, to induce a greater reliance on a complete syntactic analysis. As part of this question we employed a contrast between sentences with a SR or an OR structure, where we might expect the greater syntactic challenge of OR sentences to show a stronger effect of plausibility on comprehension responses than responses based on literal syntax. As before, the critical condition for separating these alternative processing strategies would be sentences in which the literal syntax and the plausibility of the utterance are in conflict.

Should one see an increase in comprehension responses that favor plausibility over literal syntax to occur with implausible sentences that express their meaning with an OR structure than an SR structure, one might expect this effect to be larger for older adults relative to young adults, and larger still for older adults with impaired hearing. This prediction would follow from findings that the comprehension of plausible OR sentences place a greater demand on working memory resources than plausible SR sentences (Just and Carpenter, 1992), with the behavioral consequences greater for older adults who begin with reduced working memory resources relative to younger adults (Carpenter et al., 1994). To test this hypothesis we also tested the working memory capacity of the participants in Experiment 2.

### Method

#### Participants

The young adult participants were 24 university students and staff (5 men, 19 women) ranging in age from 18 to 27 years (*M* = 19.7, *SD* = 1.94 years), all of whom had age-normal hearing acuity, as measured by PTA averaged over 500, 1,000, 2,000, and 4,000 Hz. (*M* = 8.5 dB HL, *SD* = 3.14). The group had a mean Shipley vocabulary score (Zachary, 1991) of 13.3 (*SD* = 2.24).

Forty-eight older adults were tested, 24 with good hearing acuity (7 men and 17 women) and 24 with a mild-to-moderate hearing loss (5 men and 19 women). We summarized individuals’ hearing acuity in terms of their better-ear PTA across.5, 1, 2, and 4 kHz, a range especially important for the perception of speech. Clinically normal hearing is defined as a PTA of less than 25 dB HL in the better ear (Hall and Mueller, 1997). The older adult group with better hearing acuity had a mean better-ear PTA of 16.8 dB HL (*SD* = 5.05), placing them within well range considered to be clinically normal for speech (PTA < 25 dB HL; Katz, 2002). The hearing-impaired group had a mean better-ear PTA of 35.8 dB HL (*SD* = 5.50), placing them in the mild-to-moderate hearing loss range (Katz, 2002). As indicated previously, this degree of loss represents the single largest group of hearing-impaired older adults (Morrell et al., 1996), the majority of whom do not regularly wear hearing aids (Kochkin, 1999; Fischer et al., 2011). None of the participants in the hearing-impaired group were regular users of hearing aids and all testing was conducted unaided. Potential participants who demonstrated unbalanced hearing (more than a 15 dB difference between ears under one or more frequencies) were excluded from participation.

**FIGURE 2:**
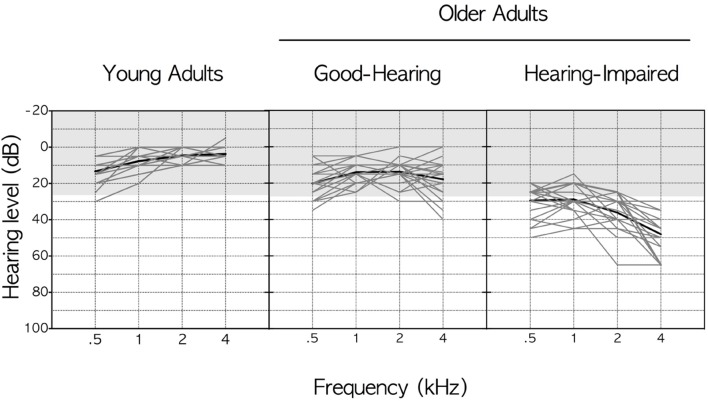
**Better-ear pure-tone thresholds from 0.5 to 4 kHz for the three participant groups.** Hearing profiles for individual listeners within each participant group are shown in light gray, with the group average shown in black. The shaded area in each of the panels indicates thresholds less than 25 dB HL.

**Figure 2** shows better-ear pure-tone thresholds from 500 to 4,000 Hz for the individual participants in the three participant groups plotted in the form of audiograms, with the *x*-axis showing the test frequencies and the *y*-axis showing the minimum sound level (dB HL) needed for their detection. Hearing profiles for individual listeners within each participant group are shown in light gray, with the group average drawn in black. The shaded area in each of the panels indicates thresholds less than 25 dB HL, a region, as indicated above, commonly considered as clinically normal hearing for speech (Katz, 2002).

The good-hearing and hearing-impaired older adults were similar in age, with the good-hearing group ranging in age from 68 to 83 years (*M* = 74.7 years, *SD* = 5.13) and the hearing-impaired group ranging in age from 69 to 81 years (*M* = 74.7 years, *SD* = 3.62). The two groups were also well-matched for verbal ability, as estimated by Shipley vocabulary scores (Zachary, 1991); older adult group with better hearing acuity, *M* = 16.3, *SD* = 2.35; hearing-impaired, *M* = 16.3, *SD* = 2.38. As is common in adult aging (Kempler and Zelinski, 1994; Verhaeghen, 2003), the older adults had somewhat better vocabulary scores than the young adults, a finding that held true for both the good-hearing, *t*(46) = 4.59, *p* < 0.001, and the hearing-impaired, *t*(46) = 4.44 *p* < 0.001, older adults. As was the case for Experiment 1, all participants reported themselves to be native speakers of American English, with no history of stroke, Parkinson’s disease, or other neurological involvement that might compromise their ability to perform the research task. None of the participants in Experiment 2 had participated in Experiment 1. Written informed consent was obtained from all participants according to a protocol approved by the Brandeis University Institutional Review Board.

##### Working memory measurement

Working memory was assessed with the Letter Number Sequencing Task (LNS; Wechsler, 1997). This is a complex span test in which participants read aloud a series of letters and numbers in sets ranging from two items to nine items, with three trials per set size. Participants are asked to repeat back the numbers first, in ascending order, followed by the letters in alphabetical order. The span measure is the total number of correct trials. This span test thus contains elements of both holding and manipulation of items in immediate memory as a measure of individual differences in working memory capacity (cf., Postle, 2006; McCabe et al., 2010).

**FIGURE 3:**
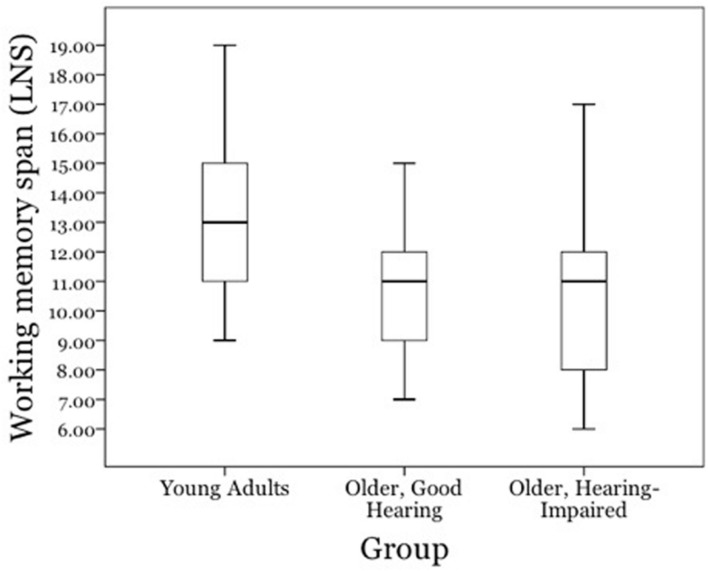
**Working memory capacity scores for young adults with age-normal hearing acuity (*young adults*), older adults with clinically normal hearing acuity for speech (*good-hearing*) and older adults with mild-to-moderate hearing loss (*hearing-impaired*)**.

**Figure 3** shows the scores of the working memory span test separately for young adults with age-normal hearing acuity (*young adults*), older adults with clinically normal hearing acuity for speech (*good-hearing*) and older adults with mild-to-moderate hearing loss (*hearing-impaired*).

Working memory scores were similar for the good-hearing (*M* = 10.8, *SD* = 2.36) and hearing-impaired (*M* = 10.3, *SD* = 3.06) older adults, *t*(46) = 0.53, n.s.). As might be expected from the body of work on adult aging and working memory (see reviews of this literature in Salthouse, 1991, 1994; Kausler, 1994), the young adults had higher working memory scores (*M* = 13.2, *SD* = 2.86) than either the older adults with better hearing acuity, *t*(46) = 3.25, *p* < 0.01, or hearing-impaired, *t*(46) = 3.36 *p* < 0.01, older adults.

#### Stimuli

Preparation of the stimuli began with construction of 64 sentences, each of which contained an action, an agent of the action, and the recipient of the action. Four versions of each sentences were then constructed: an SR version with a plausible action (e, g., “The eagle that attacked the rabbit was large”), an SR sentence with the agent and recipient switched to yield an implausible action (e.g., “The rabbit that attacked the eagle was large”), the plausible version presented with an OR sentence structure (e.g., “The rabbit that the eagle attacked was large”), and an OR sentence with an implausible action (e.g., “The eagle that the rabbit attacked was large”).

The SR and OR sentences in both their plausible and implausible versions contained exactly the same words, differing only in word order. In this example of a plausible SR sentence one can see that the main clause (*the eagle was large*) is interrupted by a relative clause (*that attacked the rabbit*). In the plausible OR sentences the embedded clause not only interrupts the main clause, but the head noun phrase (*the rabbit*) functions as both the subject of the main clause (*large*) and the object of the relative clause (*that attacked the rabbit*). Implausible OR sentences followed the same principle in which the head noun phrase serves as both the subject of the main clause and the object of the relative clause.

There are a number of reasons why comprehension of sentences with an OR structure are more challenging than sentences with an SR structure. For example, because the order of thematic roles in OR constructions are not canonical, such sentences require a more extensive thematic integration than required for the more canonical structure represented by SR sentences (Warren and Gibson, 2002). In addition, to determine these thematic roles, one must keep the subject of the sentence in mind for a longer period of time than in SR sentences (Cooke et al., 2002), such that OR constructions are thought to tax working memory to a greater degree than SR sentences (Ferreira et al., 1996; Cooke et al., 2002).

Although different authors may give different weight to each of these factors it is well-established that OR sentences result in more comprehension errors than SR sentences (Just and Carpenter, 1992; Wingfield et al., 2006), that comprehension of OR sentences are accompanied by increased patterns of neural activation in functional imaging studies (Just et al., 1996; Cooke et al., 2002; Peelle et al., 2004, 2010), and that they produce slower self-pacing patterns than SR sentences for both written (Stine-Morrow et al., 2000) and spoken (Waters and Caplan, 2001; Fallon et al., 2006) sentences.

In addition to the 256 experimental sentences (64 base sentences × 4 versions of each), 72 SR and OR filler sentences were constructed to avoid a uniform pattern of plausible and implausible non-reversible sentences. For thus purpose filler sentences were included in which the agent and recipient could be exchanged without affecting plausibility (e.g., “The boy that pushed the girl was mean”; “The boy that the girl pushed was mean”).

The experimental and filler sentences were recorded onto computer sound files by a female speaker of American English at a natural speaking rate of approximately 165 wpm and equalized within and across sentence types for RMS intensity as described in Experiment 1.

#### Procedure

Each participant heard a total of 64 experimental sentences (16 SR-plausible, 16 SR-implausible, 16 OR-plausible, 16 OR-implausible) plus 36 filler sentences. No version of any base sentence was heard more than once by any participant, with the particular base sentence heard in each of its versions counterbalanced across participants such that, by the end of the experiment, each base sentence had been heard in each of its versions an equal number of times. Stimuli were presented in a mixed-list design, with experimental sentences and filler sentences intermingled in a pseudo-random order across lists. Along with 36 filler sentences this resulted in a total of 100 sentences heard by each participant.

Instructions were the same as in Experiment 1, with participants told that following each sentence there would be a 250 ms pause, followed by a spoken probe question in the form of “Who was the do-er?” or “Who was the receiver?” Responses were to be given aloud as accurately as possible and were recorded for later scoring for accuracy.

Participants were tested individually in a sound-attenuated testing room, with stimuli presented binaurally through calibrated Eartone 3A insert earphones (E-A-R Auditory Systems, Aero Company, Indianapolis, IN, USA), via a GSI-61 audiometer (Grason-Stadler, Madison, WI, USA) at 65 dB HL. The main experiment was preceded by a brief practice session to familiarize participants with the task and the sound of the speaker’s voice.

##### Audibility Check

As in Experiment 1 a pretest was conducted in order to insure that the speech materials would be audible for all participants. This again consisted of one- and two-syllable common nouns presented one at a time at the same intensity level as would be used for the main experiment. After the presentation of each word participants were asked to repeat the word. All participants in the three participant groups showed good accuracy, with a mean of 98.3% words correctly repeated for the young adults, 96.4% correct for older adults with good hearing and 95.6% correct for older adults with a hearing impairment.

### Results

**FIGURE 4:**
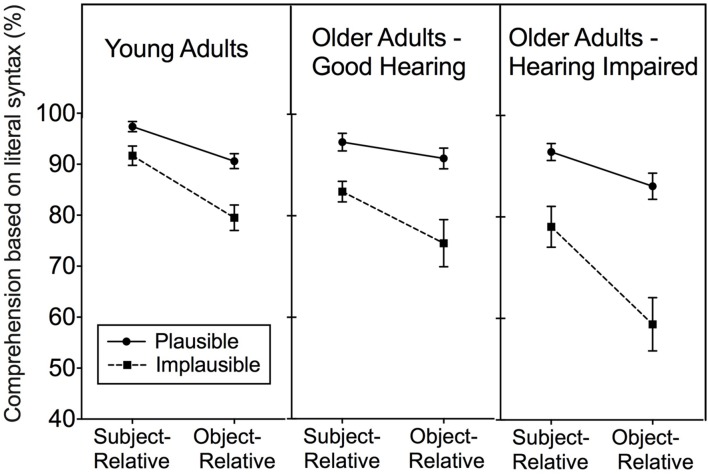
**Percentage of comprehension responses based on literal syntax represented in sentences with a subject-relative (SR) or object-relative (OR) syntactic structure when the sentence meanings were plausible or implausible.** Data for young adults with age-normal hearing, older adults with good hearing for their ages and age-matched older adults with mild-to-moderate hearing impairment are shown in the left, middle, and right panels, respectively. Error bars represent one standard error.

The left panel of **Figure 4** shows the mean percentage of times that the young adults used the literal syntax to determine who was the agent or the recipient of the action for plausible and implausible SR and OR sentences. The middle and right panels show these data for the good-hearing and hearing-impaired older adults, respectively.

There was again no difference depending on whether the agent or the recipient of the action was requested. For all analyses data were thus collapsed across the types kinds of probe questions.

The data shown in the three panels of **Figure 4** were examined with a 2 (Plausibility: plausible, implausible × 3 (Participant group: young adults, good-hearing older adults, hearing-impaired older adults) × 2 (Syntactic complexity: SR, OR) mixed design ANOVA, with plausibility and syntax as within-participants variables and groups as a between-participants variable. As implied by visual inspection of **Figure 4** there was a significant main effect of sentence plausibility, with plausible sentences more likely to produce comprehension responses consistent with the their literal syntax than for implausible sentences, *F*(1,69) = 75.58, *p* < 0.001, $\eta_{p}^{2}$ = 0.52. There was also a main effect of participant group, *F*(2,69) = 7.04, *p* < 0.01, $\eta_{p}^{2}$ = 0.17. This main effect, however, was moderated by a significant Participant group × Plausibility interaction, *F*(2,69) = 5.03, *p* < 0.01, $\eta_{p}^{2}$ = 0.13. This interaction can be seen to reflect the observation in **Figure 4** that the hearing-impaired older adults were less likely than the other two participant groups to produce comprehension responses based on the literal syntax of a sentence when the meaning of the sentence was implausible than when the meaning was plausible.

Unlike the active-passive contrast in Experiment 1, the more challenging contrast represented by SR vs. OR sentences in the present experiment now yielded a significant main effect of syntactic complexity, *F*(1,69) = 59.70, *p* < 0.001, $\eta_{p}^{2}$ = 0.46. A significant Plausibility × Syntactic complexity interaction, *F*(1,69) = 18.52, *p* < 0.001, $\eta_{p}^{2}$ = 0.21, confirmed the appearance in **Figure 4** that the effect of plausibility across the three groups was generally greater for OR sentences than for SR sentences. Neither the remaining two-way nor the three-way interactions reached significance.

The meaning of this pattern of main effects and interactions was further explored by conducting separate 2 (Plausibility) × 2 (Syntactic complexity) repeated measures ANOVAs on the data for each of the participant groups. For each of the participant groups the reduced likelihood of comprehension responses being based on the literal syntax when the sentences were implausible rather than plausible was supported by a significant main effect of plausibility (*p* < 0.001 in all cases). Each of the three groups also revealed a main effect of syntax (young adults, *p* < 0.001; good-hearing older adults, *p* < 0.05; hearing-impaired older adults, *p* < 0.001). The tendency for comprehension responses to be less likely to correspond with the literal syntax of the sentence for implausible OR sentences than for SR sentences resulted in significant Plausibility × Syntax interactions for the young adults (*p* < 0.05) and hearing-impaired older adults (*p* < 0.01), and a marginal effect for the good-hearing older adults (*p* = 0.07). An ANOVA confirmed the appearance of similar performance for all groups in the SR Plausible condition *F*(2,69) = 2.36, *p* = 0.10, confirming that the performance declines for implausible OR sentences were not due to the two older adult groups being unable to hear the stimuli as well as the young.

#### Age and Hearing as Continuous Variables

The relatively greater difficulty older adults’ have in comprehending sentences with complex syntax as compared to young adults has been attributed by many theorists to a reduced working memory capacity depriving older adults of the resources needed to support comprehension (cf., Carpenter et al., 1994; Daneman and Merikle, 1996; Caplan et al., 2011). One might thus expect that individual differences in working memory capacity would contribute significantly to the variance observed in the comprehension data.

We conducted a linear mixed-effect model regression analysis considering syntax and plausibility as categorical factors and working memory, hearing acuity, and age as continuous variables where subjects were entered as random effects. This analysis showed working memory to account for a significant amount of the variance, *t*(68) = 2.59, *p* = 0.012, and a marginal plausibility by hearing acuity interaction, *t*(210) = 1.96, *p* = 0.051. To look more closely at these effects we conducted hierarchical multiple regressions for each of the four stimulus types (SR-plausible, SR-implausible, OR-plausible, and OR-implausible), with the percentage of responses that were consistent with the literal meaning of the sentences serving as the dependent variable in each case. Predictor variables were entered into the model in the following order: working memory span represented by Letter-number Sequencing, hearing acuity represented by better-ear PTA averaged over 500, 1000, 2000, and 4000 Hz, and participants’ chronological age. This order was selected so as to determine the extent of a potential contribution of hearing acuity after statistically controlling for working memory span, and whether chronological age contributed additional variance after accounting for working memory span and hearing acuity. For each predictor variable for each of the sentence types we show *R*^2^, which represents the cumulative contribution of each variable along with the previously entered variables, and the change in *R*^2^, which shows the contribution of each variable at each step. The next column shows the level of significance of each variable and the final column shows the unstandardized regression coefficients (β).

Inspection of **Table 1** shows the prediction for working memory to be born out: Working memory scores accounted for a significant proportion of the variance in comprehension responses across all sentence conditions, albeit at a marginal level for implausible SR sentences.

**Table 1:** Summary of hierarchical regressions.

Condition	Predictor	*R*^2^	Change in *R*^2^	*p*-value	β
Subject relative plausible	Working memory	0.10	0.10	=0.01	0.61
	Hearing acuity	0.11	0.01	=0.31	-0.05
	Age	0.11	0.00	=0.62	-0.02
Subject relative implausible	Working memory	0.04	0.04	=0.08	0.30
	Hearing acuity	0.14	0.09	=0.01	-0.26
	Age	0.15	0.01	=0.32	-0.09
Object relative plausible	Working memory	0.24	0.24	=0.001	1.82
	Hearing acuity	0.25	0.02	=0.22	-0.31
	Age	0.33	0.08	=0.01	0.16
Object relative implausible	Working memory	0.10	0.10	=0.01	1.66
	Hearing acuity	0.22	0.12	=0.001	-0.83
	Age	0.23	0.01	=0.35	0.12


Although the pretest confirmed that stimuli were audible for all three participant groups, it is likely that this perceptual success came at the cost of greater perceptual effort than for those with poorer hearing acuity. This raises the concern that perceptual success in the face of reduced hearing acuity may draw cognitive resources that might otherwise be available for downstream comprehension operations, with this effect being especially damaging for more challenging sentence conditions (Pichora-Fuller, 2003; Wingfield et al., 2006). Consistent with this argument, the regression analyses in **Table 1** show hearing acuity to have contributed significantly to comprehension responses for the implausible sentences, where the literal syntax and plausibility were in conflict, but not the plausible sentences in which the two were mutually supportive.

Finally, it can also be seen that when the contributions of working memory span and hearing acuity were taken into account chronological age did not in most cases contribute additional variance to comprehension responses. We do not have an account for the singular exception for sentences in the plausible OR condition.

## General Discussion

It is reasonable to accept the generality that successful comprehension of spoken (or written) sentences rests on determination of the semantic relationships among the words of a sentence, and that these relationships are carried by the syntactic structure of the utterance (Chomsky, 1965, 1995; Frazier and Fodor, 1978; MacDonald et al., 1994). It is our contention, and that of others (e.g., Ferreira et al., 2001; Sanford and Sturt, 2002; Ferreira, 2003; Ferreira and Patson, 2007; Padó et al., 2009; Gibson et al., 2013), however, that a full syntactic analysis of the utterance is not necessarily obligatory for accurate sentence comprehension.

Evidence for this latter contention can be found in the way individuals will “hear” the missing word “to” in the sentence, “The mother gave the candle the daughter” (Gibson et al., 2013). Such examples reflect the experience-based assumption that many of the utterances we hear will be fragmentary, will have underspecified syntax, or occasional will have some words masked by background noise (cf., Goldman-Eisler, 1968; Elsness, 1984; Thompson and Mulac, 1991; Levy, 2008; Padó et al., 2009; Gibson et al., 2013).

Because we expect that the utterances we hear will have plausible meanings, one can conduct a resource-conserving shallow analysis of the sentence input, sampling some words, inferring others, and guiding our solution to the comprehension task by presumed plausibility. While the occurrence of shallow processing will go unnoticed when it results in correct comprehension, its consequences appear when errors are made. One notable example is the “Moses illusion,” in which listeners will often answer “Two” in response to the question, “How many animals of each sort did Moses put on the ark? (Erickson and Matteson, 1981; Van Oostendorp and De Mul, 1990; Van Oostendorp and Kok, 1990).

Error-free performance in the usual case of plausible sentences can obscure the role of plausibility in this success. As we have seen, however, the importance of plausibility can be revealed when the literal syntax of a sentence and its semantic plausibility are placed in conflict. We saw this in Experiment 1, where for both active-declarative and passive sentences fewer responses followed the literal meaning of the sentence when this meaning was implausible. Findings such as these are often interpreted as reflecting an age-related decline in comprehension ability for implausible sentences, with comprehension responses that favor plausibility taken as evidence for such a deficit (Obler et al., 1991; see also Yoon et al., 2015). By contrast, we would see such data, to include our own, as representing not an incorrect response but rather, as evidence of an alternative, and ordinarily adaptive, solution to the comprehension challenge.

Our finding that syntactic complexity had little effect in Experiment 1 is consistent with other studies showing a small if any effect of an active-passive manipulation (cf., Obler et al., 1991; Ferreira, 2003; Gibson et al., 2013). We introduced Experiment 1 to define the lower bounds of a syntactic effect. In Experiment 2, we contrasted SR vs. OR sentences, a contrast that has been reliably shown in numerous studies to yield significant differences in comprehension accuracy, and especially so for older adults (e.g., Just and Carpenter, 1992; Carpenter et al., 1994; Cooke et al., 2002; Wingfield et al., 2003; Wingfield et al., 2006; Peelle et al., 2010). This condition allowed a test of the hypothesis that listeners will more often engage in a resource-conserving shallow processing strategy when detection of thematic roles in an utterance via a full analysis of each word’s contribution to the sentence meaning is made more difficult by using an OR sentence structure.

Experiment 2 yielded three key findings. First, listeners’ comprehension responses were less likely to correspond to the literal meanings of the utterances if this process yielded an implausible meaning. Second, the ratio of comprehension responses based on the meaning as determined by the literal syntax relative to responses that opted for a plausible interpretation when the two were in conflict, was larger with the less syntactically demanding SR sentences than the more resource-demanding OR sentences. Finally, this effect was markedly greater for the older adults with a mild-to-moderate hearing loss, all of whom passed an audibility screen for speech presented at the same sound intensity as used in the main experiment. This should not imply, however, that their success did not come at the cost of greater perceptual effort than for the young adults or the good-hearing older adults. When hearing acuity was taken as a continuous variable in the regression analysis we saw that hearing acuity did indeed add to the variance in comprehension responses for the implausible sentences, where syntax and plausibility were in conflict, but not for the plausible sentences where the two were in accord.

Two final caveats should be mentioned. In the first case, in the absence of a real-time measure of processing operations we cannot say whether syntactic parsing, determination of semantic relations within the sentence and testing against real-world plausibility are processed concurrently, or whether one conducts a syntax-first analysis followed by a plausibility check after the initial-phase processing has been completed (for a discussion see Padó et al., 2009).

Our experimental task is intended to represent effects of syntax and plausibility in sentence comprehension (e.g., Ferreira, 2003; Ferreira and Patson, 2007). It should be acknowledged, however, that plausibility could have exerted its effect at the time that the comprehension question probe was delivered. Whichever is the case, however, it is clear from that listening effort consequent to age-related hearing loss leads to greater reliance on plausibility in these data than for either age-matched older adults with good-hearing acuity or, in turn, younger adults with age-normal hearing.

Second, it should be acknowledged that perceptual or cognitive effort in listening tasks are most often assessed, as was the case here, as a performance decline for degraded but audible speech vs. clearer speech (e.g., Rabbitt, 1968, 1991; Surprenant, 1999, 2007; Pichora-Fuller, 2003; Pichora-Fuller and Souza, 2003; McCoy et al., 2005; Wingfield et al., 2006). Attempts to find a measure of processing effort independent of performance on the target task itself have included reduced accuracy on a concurrent non-language secondary task while listening to and recalling clear vs. degraded speech (e.g., Larsby et al., 2005; Sarampalis et al., 2009; Tun et al., 2009; Fraser et al., 2010), an increase in pupil dilation of the eye while listening to degraded speech as an indicator of effortful processing (Zekveld et al., 2011; Kuchinsky et al., 2013) and increased patterns of neural activation revealed in functional neuroimaging (Peelle et al., 2010, 2011). It remains the case, however, that that the cognitive literature has yet to reach a consensus on a formal definition of effort or effortful processing (for a discussion of attempts, see McGarrigle et al., 2014).

## Conclusion and Future Directions

It has been argued that a goal of cognitive aging research should be removal of chronological age as an experimental variable (e.g., Kausler, 1994). We attempted to follow this goal in Experiment 2, with regression analyses showing that for the present task once working memory and hearing acuity were taken into account, in all but one sentence condition chronological age did not add additional variance to the nature of the comprehension response. The three factors we considered (working memory capacity, hearing acuity, and age), however, still left considerable variance unaccounted for that might be accounted for by additional variables not tested. One possible candidate may be individual differences in self-efficacy and control beliefs that can affect performance in a number of domains (cf., Lachman and Jelallian, 1984; Hastings and West, 2011; Smith et al., 2011; Agrigoroaei et al., 2013). We suggest this as a fruitful area for future research.

## Author Contributions

NA and AW contributed equally to the design, and conduct of the research and in preparation of this manuscript. AGW contributed to the conduct of experiment 1.

## Conflict of Interest Statement

The authors declare that the research was conducted in the absence of any commercial or financial relationships that could be construed as a potential conflict of interest.

The reviewer CS and handling Editor declared their shared affiliation, and the handling Editor states that the process nevertheless met the standards of a fair and objective review.
